# MRI features and tumor-infiltrating CD8 + T cells-based nomogram for predicting meningioma recurrence risk

**DOI:** 10.1186/s40644-024-00731-6

**Published:** 2024-06-28

**Authors:** Tao Han, Xianwang Liu, Changyou Long, Shenglin Li, Fengyu Zhou, Peng Zhang, Bin Zhang, Mengyuan Jing, Liangna Deng, Yuting Zhang, Junlin Zhou

**Affiliations:** 1 Department of Radiology, Lanzhou University Second Hospital, Lanzhou, 730000 China; 2 Second Clinical School, Lanzhou University, Lanzhou, 730000 China; 3 Key Laboratory of Medical Imaging of Gansu Province, Lanzhou, 730000 China; 4 Gansu International Scientific and Technological Cooperation Base of Medical Imaging Artificial Intelligence, Lanzhou, 730030 China; 5 Image Center of Affiliated Hospital of Qinghai University, Xining, 810001 China; 6 Department of Pathology, Lanzhou University Second Hospital, Lanzhou, 730000 China

**Keywords:** Meningioma, Histogram analysis, Magnetic resonance imaging, Recurrence, Tumor microenvironment

## Abstract

**Objective:**

This study was based on MRI features and number of tumor-infiltrating CD8 + T cells in post-operative pathology, in predicting meningioma recurrence risk.

**Methods:**

Clinical, pathological, and imaging data of 102 patients with surgically and pathologically confirmed meningiomas were retrospectively analyzed. Patients were divided into recurrence and non-recurrence groups based on follow-up. Tumor-infiltrating CD8 + T cells in tissue samples were quantitatively assessed with immunohistochemical staining. Apparent diffusion coefficient (ADC) histogram parameters from preoperative MRI were quantified in MaZda. Considering the high correlation between ADC histogram parameters, we only chose ADC histogram parameter that had the best predictive efficacy for COX regression analysis further. A visual nomogram was then constructed and the recurrence probability at 1- and 2-years was determined. Finally, subgroup analysis was performed with the nomogram.

**Results:**

The risk factors for meningioma recurrence were ADCp1 (hazard ratio [HR] = 0.961, 95% confidence interval [95% CI]: 0.937 ~ 0.986, *p* = 0.002) and CD8 + T cells (HR = 0.026, 95%CI: 0.001 ~ 0.609, *p* = 0.023). The resultant nomogram had AUC values of 0.779 and 0.784 for 1- and 2-years predicted recurrence rates, respectively. The survival analysis revealed that patients with low CD8 + T cells counts or ADCp1 had higher recurrence rates than those with high CD8 + T cells counts or ADCp1. Subgroup analysis revealed that the AUC of nomogram for predicting 1-year and 2-year recurrence of WHO grade 1 and WHO grade 2 meningiomas was 0.872 (0.652) and 0.828 (0.751), respectively.

**Conclusions:**

Preoperative ADC histogram parameters and tumor-infiltrating CD8 + T cells may be potential biomarkers in predicting meningioma recurrence risk.

**Clinical relevance statement:**

The findings will improve prognostic accuracy for patients with meningioma and potentially allow for targeted treatment of individuals who have the recurrent form.

**Supplementary Information:**

The online version contains supplementary material available at 10.1186/s40644-024-00731-6.

## Key points

• Currently available methods for determining meningioma recurrence are inaccurate.

• CD8 + T cells counts successfully predicted meningioma recurrence.

**Supplementary Information:**

The online version contains supplementary material available at 10.1186/s40644-024-00731-6.

## Introduction

Meningiomas are common, slow-growing, primary tumors that originate in arachnoid capillary cells and account for 39.0% of all central nervous system (CNS) tumors [1]. Although more than 80% of meningiomas are benign, a subset become aggressive and are categorized by the World Health Organization (WHO) as grade 2 to 3 meningiomas. These are difficult to completely eradicate surgically, and patients experience early or multiple tumor recurrences. Treatments vary depending on meningioma grade. Grade 1 tumors are monitored, whereas grade 3 tumors require radiotherapy. The treatment for patients with grade 2 meningiomas is less universal; total resection remains controversial regardless of whether the procedure is accompanied by radiotherapy or observation [2]. Partially resected grade 2 tumors are treated with adjuvant radiotherapy. Unfortunately, the 10-year progression-free survival (PFS) rates for grades 1, 2, and 3 meningiomas are 75–90%, 23–78%, and 0%, respectively, reflecting the relative ineffectiveness of available treatments. Indeed, the development of systemic therapies (e.g., immunotherapy) for refractory meningioma is a major clinical challenge for postoperative management [3].

One clear obstacle is the difficulty in predicting meningioma recurrence. The tumor microenvironment (TME) consists of numerous cell types and a variable extracellular matrix that enhance tumor immune tolerance, directly influencing cancer progression and recurrence [4]. A previous study had shown that meningiomas have a large macrophage infiltrate [5]. Tumor-infiltrating lymphocytes (TILs) are important components of the meningioma TME and have a major effect on underlying tumor growth, proliferation, and overall prognosis [6]. In particular, CD8 + TILs exert antitumor effects [7], and their decrease in anaplastic meningiomas suggests the presence of an immunosuppressive tumor microenvironment.

Because meningiomas are intracranial, extracerebral tumors and totally located outside the blood-brain barrier, they are fed by both the meningeal and cerebral arteries. This access to circulating cells underlies their immune infiltrative characteristics [8]. A previous study showed that WHO meningioma grades are negatively correlated with the proportion of CD4+, CD8+, and PD-1 + lymphocytes [9]. One study demonstrated that CD8 + TIL levels correlate with meningioma recurrence and that grade 2–3 tumors are in a state of relative immunosuppression [3]. For immunotherapy to become a realistic therapeutic approach, TILs in meningiomas must be thoroughly characterized, but few studies are available with the necessary data.

Currently, meningioma is mainly diagnosed by radiologists through preoperative MRI. Conventional MRI features, such as tumor location and diameter were thought to be associated with meningioma recurrence [10, 11]. However, these qualitative analyses are limited in predicting meningioma recurrence/progression and risk stratification. In contrast, histogram analysis is a multiparameter image-processing tool that can reflect cellular heterogeneity and microstructural data via extracting first-order histogram features from within a region of interest (ROI) [12]. Apparent diffusion coefficient (ADC) histogram analysis is now widely used for CNS tumors and has excellent outcomes in meningioma grading, subtyping, and differential diagnosis [13, 14]. However, we have little data regarding whether ADC histogram parameters can predict meningioma recurrence preoperatively.

Therefore, this study aimed to investigate the prognostic value of preoperative MRI features (specifically ADC histogram parameters) and tumor-infiltrating CD8 + T cells in risk stratification for meningioma recurrence.

## Materials and methods

### Patients

This retrospective study was approved by the Ethics Review Committee of our institution (2023 A-169) and the need for informed consent was waived. Preoperative clinical, MRI, and postoperative pathological datas of patients with histopathologically confirmed diagnosis of meningioma from January 2019 to June 2022 in our hospital were retrospectively analysed. Inclusion criteria: (1) resection biopsy specimens were histopathologically confirmed to be meningiomas and immunohistochemical staining for CD8 was available; (2) all patients underwent meningioma resection 1 week after MRI; (3) complete clinical, imaging, and pathological data. Exclusion criteria: (1) patients received radiotherapy, targeted therapy, or other treatments before preoperative MRI scanning; (2) incomplete image sequences or failure to meet the diagnostic requirements; (3) patients with tumor diameter < 1 cm; (4) patients lost to follow-up; and (5) unable to obtain tissue specimens after surgical resection. Finally, a total of 102 patients with meningiomas were included in this study. The flow diagram of patient inclusion and exclusion as showed in Supplementary Material Fig S1.

### Follow-up

The primary endpoint was tumor recurrence, defined as the time interval between initial diagnosis (surgery date) and recurrence date. All enrolled patients were followed-up with MRI scans until August 31, 2023. Recurrence was defined as a new lesion in the operated area or a > 10% increase in residual lesion volume on MRI T1C at any time during the follow-up period; otherwise, the patient was categorized as non-recurrence.

### MRI scanning protocol

All enrolled patients underwent preoperative MRI examinations (T1-weighted image (T1WI), T2-weighted images (T2WI), DWI, and T1C), using a 3.0-T MRI Scanner (Siemens Verio, Erlangen, Germany) with an 8-channel head coil. The scanning parameters were as follows: (1) gradient echo sequences: T1WI ( repetition time [TR] = 550 ms, time to echo [TE] = 11 ms), slice thickness 5 mm, slice spacing 1.5 mm, field of view (FOV) 260 mm × 260 mm, and matrix 269 × 384; (2) turbo spin echo: T2WI (TR = 2200 ms, TE = 96 ms), echo time 10 ms, and two excitations, and matrix 256 × 256; (3) DWI: (TR = 4500 ms, TE = 102 ms), slice thickness 5 mm, slice spacing 1.5 mm, FOV 260 mm × 260 mm, and matrix 160 × 160, and b values of 0 and 1000 s/mm2; and (4) T1C: gadolinium diethyltriaminopentylacetate (0.1 mmol/kg) was injected through the elbow vein at a rate of 3 mL/s with the same scanning parameters as those for T1WI, and axial, coronal, and sagittal images were obtained.

### Analysis of conventional MRI features

The conventional MRI features of all enrolled meningioma patients were independently evaluated by two radiologists (radiologists 1 and 2, with 10 and 8 years of experience in diagnostic neuroradiology, respectively) using a double-blind method to assess the following conventional MRI features: (1) tumor location: skull base, non-skull base; (2) necrosis: present, absent; (3) tumor enhancement: homogeneous, inhomogeneous; (4) tumor shape: round, lobulation; (5) tumor maximum diameter; (6) tumor volume (VT = 4πabc/3); (7) peritumour edema diameter; (8) peritumour edema volume (VE = VT2-high - VT); and (9) edema index (EI = (VE + VT)  / VT). Two experienced neurosurgeons, unaware of the imaging datas, retrospectively analysed the Simpson grading of the patients who underwent meningioma resection from the PACS operative records.

### ADC histogram analysis

Preoperative ADC images of all enrolled patients were obtained from DWI images after post-processing, and all ADC images were imported into MaZda software (version 4.6, Technical University of Lodz, Institute of Electronics, Łódz, Poland, http://www.eletel.p.lodz.pl/mazda/) after adjusting the window width and window position. Then the largest level of the tumor was selected on the axial ADC images, and the tumor boundary was determined by T1C images, and ROIs were manually sketched along the edges of the tumor on the axial ADC images by the two radiologists mentioned above. In order to better assess the heterogeneity of the tumor, ROIs included cystic and hemorrhage areas of the tumor, but excluded peritumoural edema areas. The sketched ROIs were filled in red and the following parameters were automatically generated: mean, variance, skewness, kurtosis, perc.01%, perc.10%, perc.50%, perc.90%, and perc.99%. Disagreements were resolved by negotiation when they occurred, and the final result was the average of two radiologist measurements.

### Pathological analysis

**Fig. 1:**
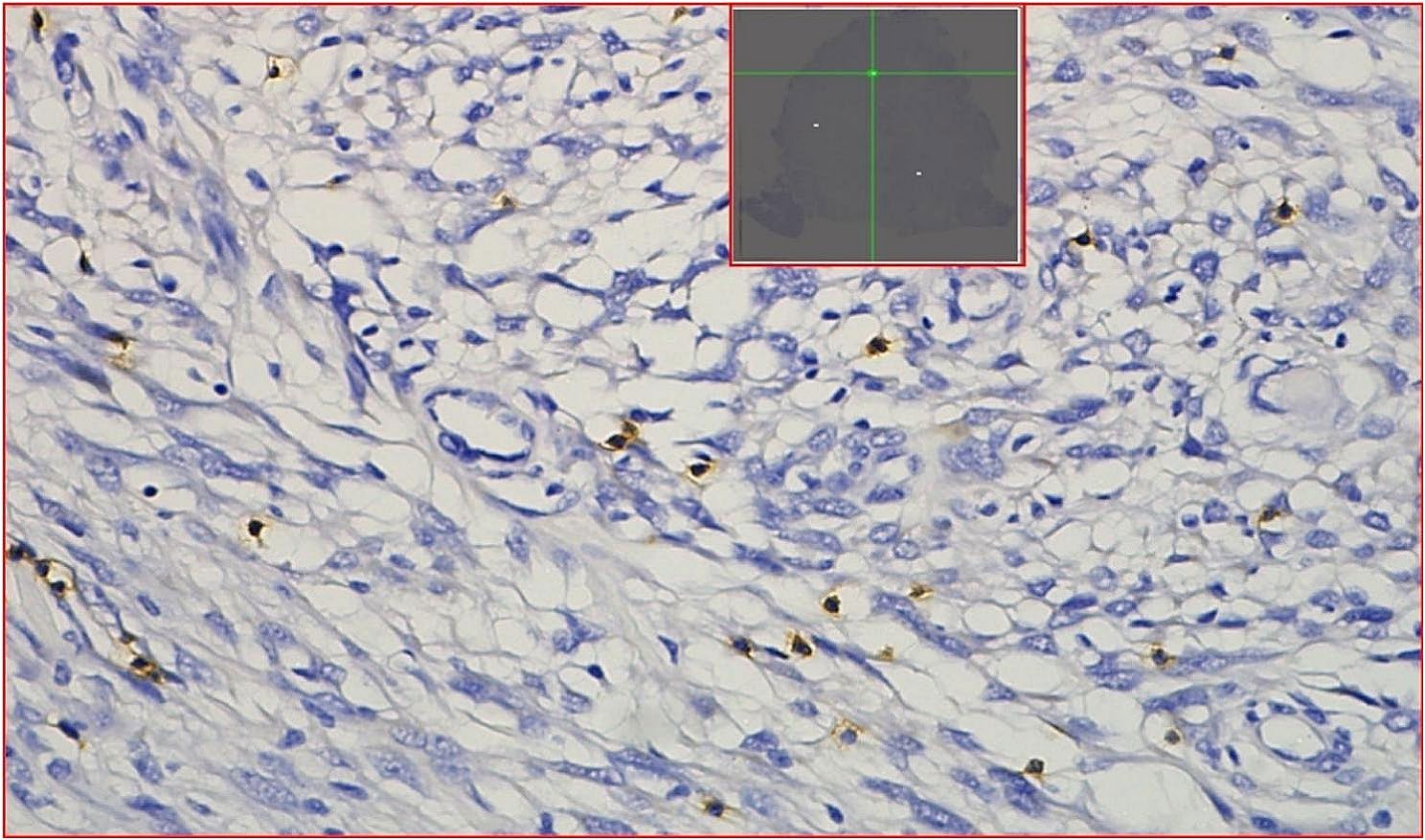
The WSI was checked by DSAssistant software, and 3 hot spots ROI images of CD8 + T cells IHC maps were randomly divided

**Fig. 2:**
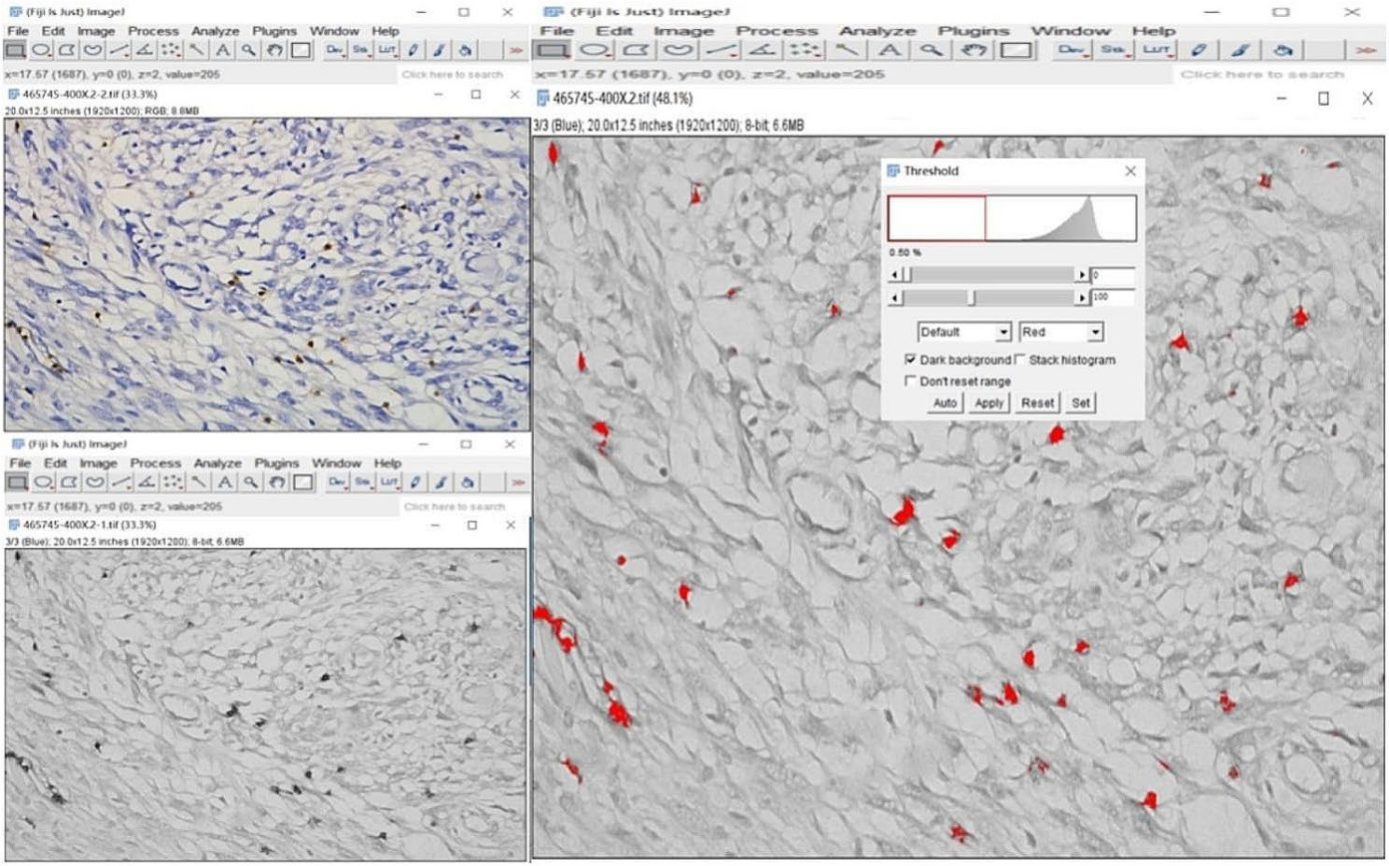
The percentage of CD8 + T cells was obtained by CD8 immunohistochemical staining and gray level transformation and threshold adjustment

CD8 immunohistochemical staining were performed in 102 patients with meningioma after surgical resection. Paraffin specimens of postoperative meningiomas were cut into 4 μm sections. The CD8 antibody (RMA-0514, 1:100; Maixin) was used for immunohistochemical staining, revealing brownish-yellow cell membranes that suggested the positive surface expression of CD8 protein. All CD8-stained sections were scanned as whole-slide images (WSI) using the Motic EasyScanner digital section scanning system and viewed in DSAssistant software. For each CD8 WSI image, three different high-power fields (HPFs, 400× magnification) were randomly selected as ROIs. Segmented ROI images were analyzed by a neuropathologist in Image J software (version ImageJ2, USA). Final values for CD8 + T cells were the average of three ROIs and expressed as percentages (%), as shown in Figs. 1 and 2.

Immunohistochemical analysis of Ki-67 was performed using a monoclonal mouse anti-human Ki-67 antibody; brownish-yellow nuclei were considered Ki-67 positive. Regions with the highest concentration of Ki-67-positive cells (number of Ki-67 antibody positives per 1,000 tumor cells) were selected for evaluation. The Ki-67 proliferation index (PI) was calculated as the number of positive cells/total cell count.

### Statistical analysis

MedCalc 19.1 (MedCalc, Mariakerke, Belgium) and R software (version 4.2.1; http://www.Rproject.org were used for statistical analysis. Categorical variables were expressed as number of cases (percentage) and were tested using the chi-square test or Fisher’s exact test. The Shapiro-Wilk test was used to test the normal distribution of continuous variables. Conventional MRI features and histogram parameters conforming to the normal distribution were expressed as mean ± standard deviation, and the differences between the groups were compared by the independent samples t-test. The non-normally distributed were expressed using the median ± quartile spacing, and the differences between the groups were compared by the Mann-Whitney U test. A multivariate COX proportional risk model was used to identify independent risk factors for meningioma recurrence and a nomogram was constructed, and validated the model to predict recurrence of WHO grade 1 and WHO grade 2 meningiomas. Recurrence-free survival curves were plotted using the Kaplan-Meier method, and differences between curves were analysed by log-rank tests. The probability of recurrence at 1-year and 2-years was assessed by plotting the receiver operating characteristic (ROC) curve. Interobserver agreement of conventional MRI characteristics and ADC histogram parameters was assessed using the intraclass correlation coefficient (ICC), with good interobserver agreement at ICC > 0.75. *p* < 0.05 was considered a statistically significant difference.

## Results

### Basic characteristics and conventional MRI features

**Fig. 3:**
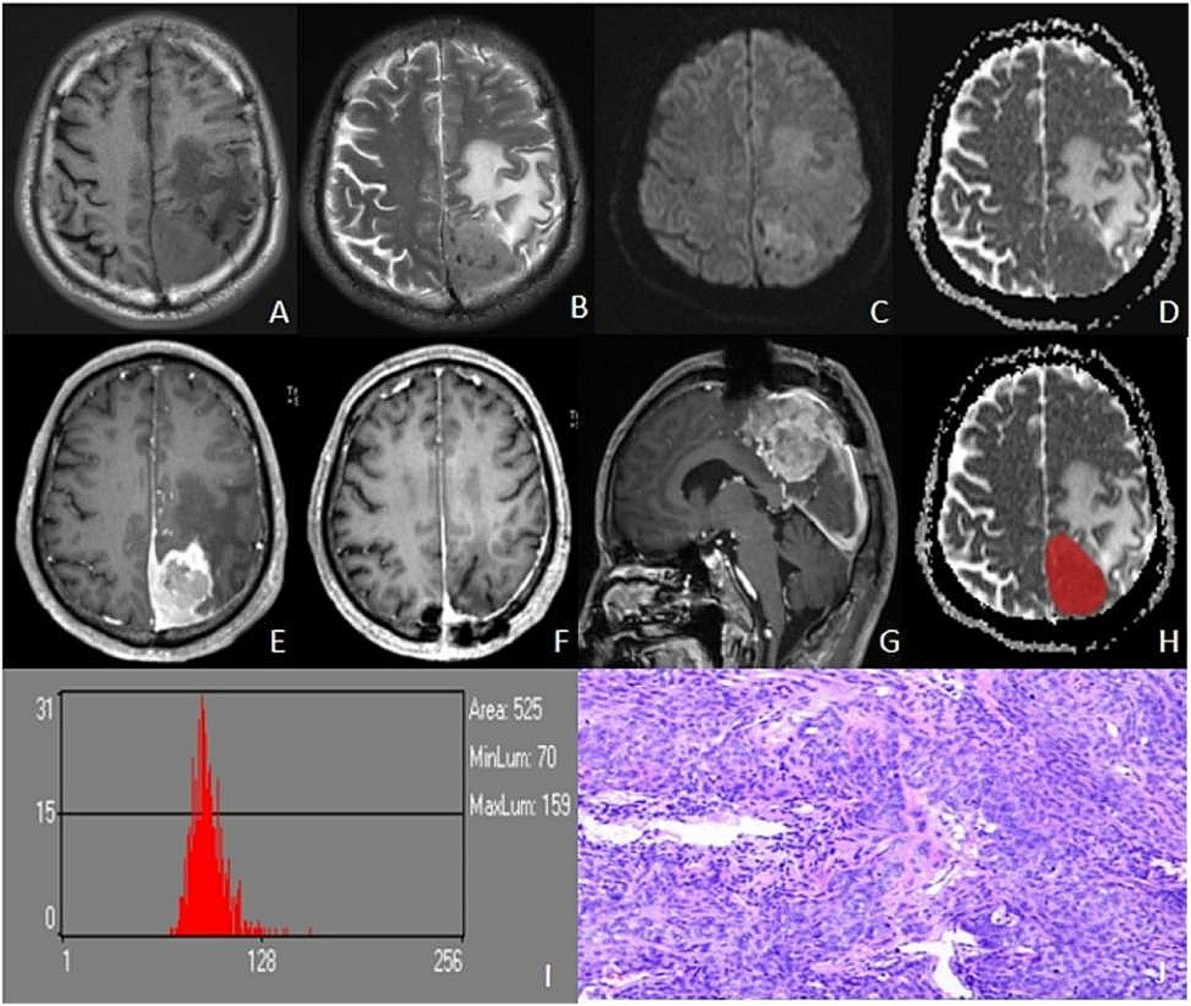
Male, 64 years old, left parietal atypical meningioma with recurrence 20 months after surgery. Irregularly shaped iso-T1WI **(A)** and iso-T2WIsignals in the left parietal, with peritumoural edema **(B)**, slightly hyperintensity in DWI **(C)**, and reduced signals in ADC **(D)**. T1C showed significant inhomogeneous enhancement **(E)**, and there was no recurrence at 3 months after resection **(F)** and 20 months tumor recurrence **(G)**. Outlined ROI **(H)**, ADC histogram parameters values were as follows: mean, 91.265; variance, 146.88; skewness, -1.3786; kurtosis, 14.874; ADCp1, 70; ADCp10, 81; ADCp50, 91; ADCp90, 103; ADCp99,130 **(I)**. Pathology (HE×100): atypical meningioma, tumor invades brain tissue, multifocal map-like coagulative necrosis, some areas of tightly arranged cells, increased nucleoplasmic proportion of cells, dark staining, pseudo daisy-shaped cluster structure arranged around blood vessels, KI-67% (10–30%) **(J)**

**Fig. 4:**
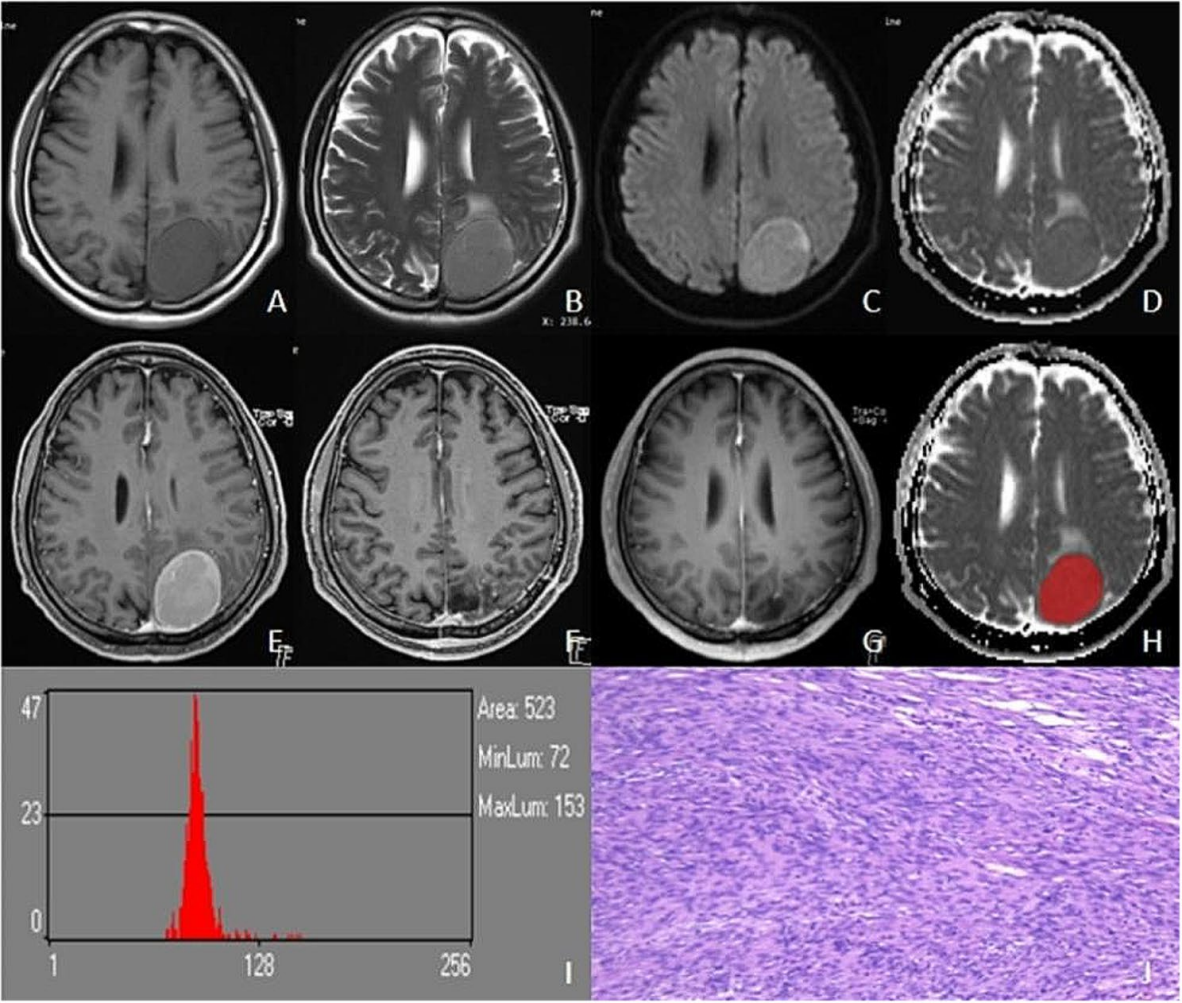
Male, 52 years old, left parieto-occipital fibroblastic meningioma with no recurrence till 28 months. The left parieto-occipital region showed a round iso T1WI **(A)** and slightly longer T2WI signal **(B)**, with signal homogeneity, slightly hyperintensity on DWI **(C)**, and iso-signal on ADC **(D)**. T1C showed significant homogeneous enhancement **(E)**, and there was no recurrence at 3-month **(F)** and 28-month postoperative review **(G)**. Outlined ROI **(H)**, ADC histogram parameters values were as follows: mean, 94.072; variance, 320.36; skewness, 4.7907; kurtosis, 30.683; ADCp1, 75; ADCp10, 83; ADCp50, 91; ADCp90, 103; ADCp99,184 **(I)**. Pathology (HE×100): fibroblastic meningioma KI-67% was 2% **(J)**

The study included 102 patients with meningioma. The recurrence group contained 30 cases (11 men and 19 women) with a mean age of 48.03 ± 14.31 years. The non-recurrence group contained 72 cases (19 men and 53 women) with a mean age of 51.99 ± 13.24 years. The two groups differed significantly (*p* < 0.05) in the conventional MRI features of enhancement, grade, and Ki-67 PI, but not in basic demographic or clinical characteristics, including sex, age, location, necrosis, tumor shape, Simpson grade, postoperative radiotherapy, tumor diameter, tumor volume, edema diameter, edema volume, and edema index (*p* > 0.05), Table 1; Figs. 3 and 4.

**Table 1:** The comparison of baseline characteristics, conventional MRI features, and CD8 + T cells in recurrence and non-recurrence meningiomas[ case (%)]

Parameters	Recurrence(*n* = 30)	Non-recurrence(*n* = 72)	Statistical values	*p*-value
Age	48.03 ± 14.31	51.99 ± 13.24	1.342	0.183
Sex			1.077	0.299
Female	19 (63.3%)	53 (73.6%)		
Male	11 (36.7%)	19 (26.4%)		
Location			0.413	0.521
Skull base	8 (26.7%)	15 (20.8%)		
Non skull base	22 (73.3%)	57 ( 79.2%)		
Necrosis			0.596	0.440
Yes	15 (50.0%)	30 (41.7%)		
No	15 (50.0%)	42 (58.3%)		
Grade			13.909	0.001
WHO 1	12 (40.0%)	54 (75.0%)		
WHO 2	16 (53.3%)	18 (25.0%)		
WHO 3	2 (6.7%)	0 (0.0%)		
Enhancement			4.870	0.027
Homogeneous	6 (20.0%)	31 (43.1%)		
Inhomogeneous	24 (80.0%)	41 (56.9%)		
Tumor shape			2.857	0.091
Round	12 (40.0%)	42 (58.3%)		
Lobulation	18 (60.0%)	30 ( 41.7%)		
Tumor diameter	4.24 ± 1.20	4.73 ± 1.27	1.798	0.075
Tumor volume	208.97 ± 253.11*	269.69 ± 367.00*	−1.447	0.148
Edema diameter	0.00 ± 3.15*	1.55 ± 3.80*	−0.912	0.362
Edema volume	48.52 ± 11.62*	56.22 ± 12.11*	−1.013	0.311
Edema index	1.00 ± 1.80*	1.00 ± 0.81*	−0.530	0.596
Ki−67 PI	5.00 ± 17.00*	3.00 ± 5.75*	−2.621	0.009
Simpson grade			2.982	0.394
Simpson I	4 (13.3%)	17 (23.6%)		
Simpson II	6 (20.0%)	19 (26.4%)		
Simpson III	11 (36.7%)	23 (31.9%)		
Simpson IV	9 (30.0%)	13 (18.1%)		
Postoperative therapy			1.772	0.183
Yes	4 (13.3%)	4 (5.6%)		
No	26 (86.7%)	68 (94.4%)		
CD8 + T cells (%)	0.06 ± 0.13*	0.17 ± 0.25*	−3.382	0.001

Continuous variables with a normal distribution are presented as mean ± standard deviation; non-normal variables are reported as median ± interquartile; number of cases (percentage) for categorical data

* non-normal distribution

### ADC histogram parameters

The mean, variance, skewness, kurtosis, and all percentile values showed excellent inter-reader agreement (ICC, 0.801–0.951). Differences in ADCp1, ADCp10, ADCp50, ADCp90, and ADCp99 were significant between the two groups, with the recurrence group having lower values (*p* < 0.05), and we found that ADCp1 had the best predictive efficacy for COX regression analysis further, Table 2.

**Table 2:** The comparison of ADC histogram parameters in recurrence and non-recurrence meningiomas [ case (%)]

Parameters	Recurrence(*n* = 30)	Non-recurrence (*n* = 72)	Statistical values	*p*-value
Mean	97.94 ± 24.44*	101.73 ± 23.35*	−1.439	0.150
Variance	137.68 ± 151.99*	131.94 ± 180.98*	−0.481	0.630
Skewness	0.86 ± 1.83*	1.26 ± 1.54*	−1.061	0.289
Kurtosis	2.49 ± 7.68*	3.08 ± 8.84*	−0.738	0.460
ADCp1	76.00 ± 14.50*	86.00 ± 17.00*	−4.016	<0.001
ADCp10	87.00 ± 14.50*	94.00 ± 20.75*	−3.130	0.002
ADCp50	97.50 ± 14.50*	106.00 ± 24.75*	−2.619	0.009
ADCp90	110.50 ± 31.25*	125.00 ± 29.50*	−2.167	0.030
ADCp99	131.50 ± 45.00*	150.00 ± 40.00*	−2.163	0.031

Continuous variables with a normal distribution are presented as mean ± standard deviation; non-normal variables are reported as median ± interquartile; number of cases (percentage) for categorical data

* non-normal distribution

### Tumor-infiltrating CD8 + T cells

Tumor-infiltrating CD8 + T cells were lowly expressed in meningiomas overall. However, the recurrence group contained significantly (*p* = 0.001) fewer CD8 + T cells (0.06 ± 0.13)% than the non-recurrence group (0.17 ± 0.25)%, as shown in Table 1.

### Univariate and multivariate COX proportional risk predictions

**Fig. 5:**
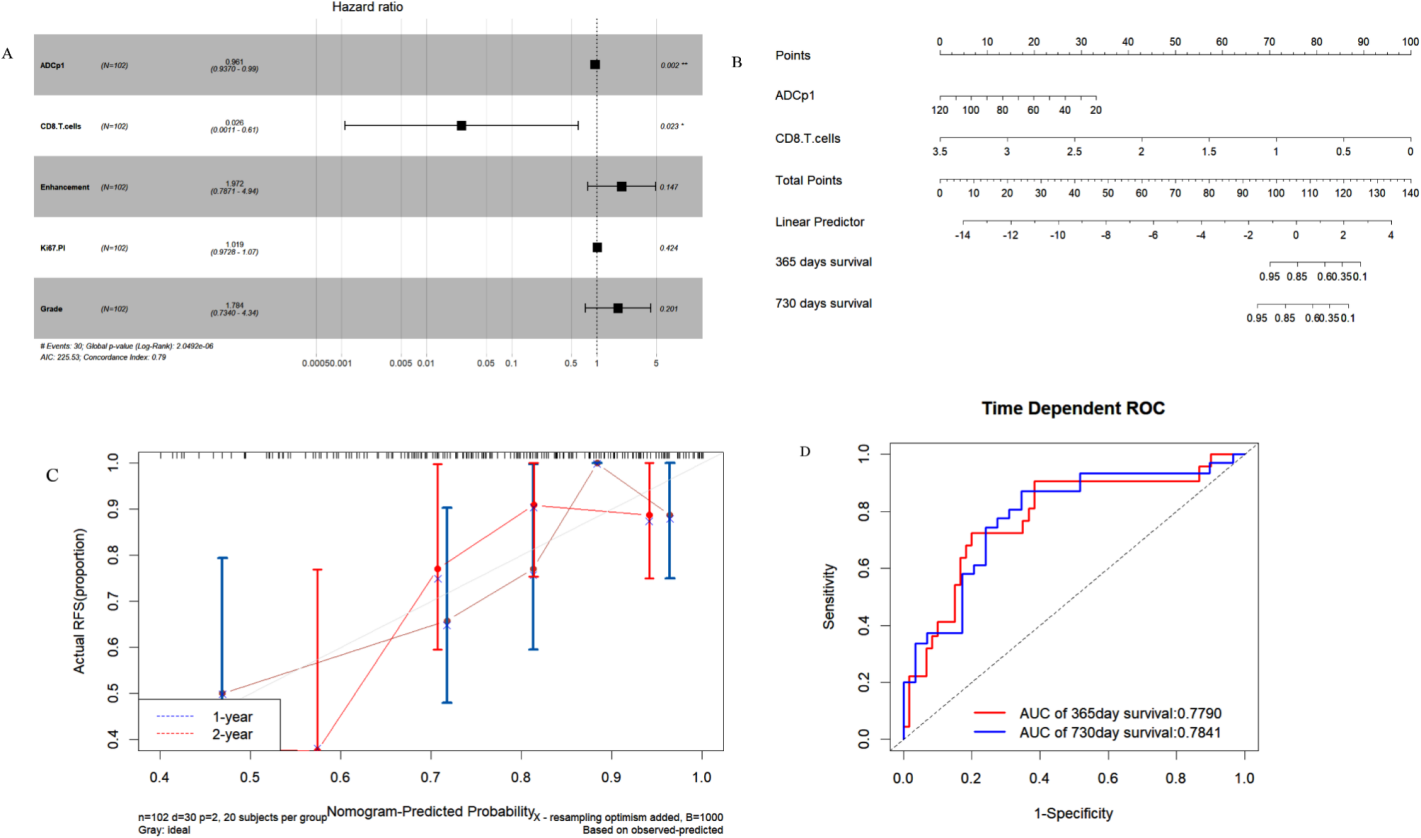
**A**: Two risk factors were screened out for constructing the nomogram. **B**: The nomogram was constructed based on Cox proportional hazards regression model, including ADCp1 and CD8 + T cells. **C**: The 1-year, 2-years calibration curves for the nomogram. **D**: The 1-year, 2-years ROC curves for the nomogram

Univariate COX regression revealed that enhancement, grade, Ki-67 PI, ADCp1, and CD8 + T cell levels were significant predictors of meningioma recurrence. Multivariate COX regression further demonstrated that ADCp1 (hazard ratio [HR] = 0.961, 95%CI = 0.937 ~ 0.986, *p* = 0.002) and CD8 + T cells (HR = 0.026, 95%CI = 0.001 ~ 0.609, *p* = 0.023) were independent risk predictors influencing recurrence (Table 3; Fig. 5A). We constructed a COX proportional hazards nomogram to predict the probability of recurrence-free survival (RFS) among patients with meningioma (Fig. 5B). Calibration curves showed that model predictions were similar to actual observations (Fig. 5C). Additionally, model AUC values were 0.779 and 0.784 for predicting 1-year and 2-year postoperative RFS, respectively (Fig. 5D).

**Table 3:** Univariate and multivariate logistic analysis of conventional MRI features, ADC histogram parameters, and CD8 + T cells in predicting meningiomas recurrence

Parameter	Univariate Analysis			Multivariate Analysis		
	HR	95%CI	*p*-value	HR	95%CI	*p*-value
Sex	1.649	0.778 ~ 3.493	0.192			
Age	0.985	0.959 ~ 1.012	0.266			
Location	0.877	0.389 ~ 1.977	0.751			
Necrosis	1.464	0.725 ~ 2.956	0.288			
Grade	3.318	1.769 ~ 6.224	*p*<0.001	1.784	0.734 ~ 4.336	0.201
Ki−67 PI	1.042	1.007 ~ 1.079	0.019	1.019	0.973 ~ 1.068	0.424
Enhancement	2.555	1.043 ~ 6.262	0.040	1.972	0.787 ~ 4.943	0.147
Tumor shape	1.999	0.961 ~ 4.159	0.064			
Simpson grade	1.374	0.954 ~ 1.980	0.088			
Postoperative therapy	2.810	0.970 ~ 8.140	0.057			
Tumor diameter	0.789	0.581 ~ 1.070	0.127			
Tumor volume	0.999	0.997 ~ 1.001	0.181			
Edema diameter	0.945	0.793 ~ 1.126	0.525			
Edema volume	1.000	0.999 ~ 1.001	0.477			
Edema Index	1.122	0.982 ~ 1.283	0.090			
ADCp1	0.960	0.941 ~ 0.979	*p*<0.001	0.961	0.937 ~ 0.986	0.002
CD8 + T cells	0.032	0.002 ~ 0.591	0.021	0.026	0.001 ~ 0.609	0.023

HR: Hazard ratio; CI: confidence interval

### Stratified survival analysis using recurrence predictors

**Fig. 6:**
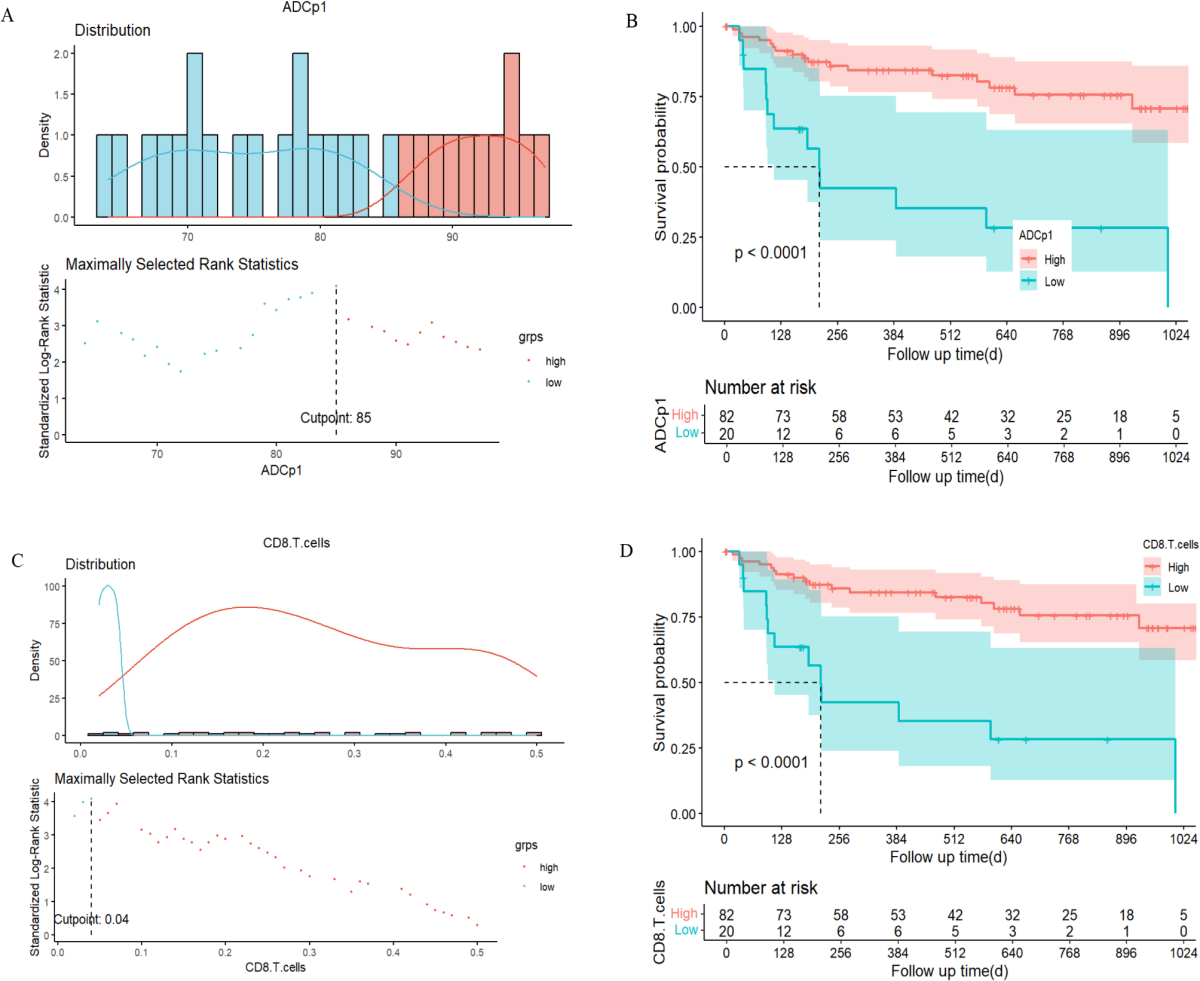
**A, B**: RFS in meningioma patients stratifed by ADCp1 = 85; **C, D**: RFS in meningioma patients stratifed by CD8 + T cells = 0.04%

Kaplan-Meier analyses demonstrated that ADCp1 (log-rank test: *p* < 0.001) and CD8 + T cells (log-rank test: *p* < 0.001) were significantly correlated with RFS. Patients with low ADCp1 (ADCp1<85) or low CD8 + T cells (CD8 + T cells<0.04) had a higher RFS rate than patients with high ADCp1 or high CD8 + T cells (Fig. 6).

### Validation of the nomogram to predict recurrence of WHO grade 1 and WHO grade 2 meningiomas

Patients with WHO grade 1, 2, and 3 meningiomas were included in this study as 66, 34, and 2 patients respectively. To determine whether the nomogram worked well in different pathologically grade meningiomas, we performed a validation analysis of WHO grade 1 and WHO grade 2 meningiomas. The results revealed that the nomogram predicted the AUC of 1-year and 2-year recurrence for WHO grade 1 and WHO grade 2 meningiomas to be 0.872 (0.652) and 0.828 (0.751) respectively, Supplementary Material Fig S2.

## Discussion

We developed a COX proportional risk prediction model for postoperative RFS in patients with meningioma. The model integrated two independent risk factors, ADCp1 and CD8 + T cells, to predict recurrence probability per patient after 1- and 2-years. The ROC curves, calibration curves, and Kaplan-Meier survival curves demonstrated that the model reliably predicted RFS in patients with meningioma, and the validity of the nomogram in WHO grade 1 and WHO grade 2 meningiomas has also been further validated. Thus, our model should provide guidance for postoperative management of patients with meningioma.

Previous studies have found that meningioma recurrence is often associated with pathological grade, KI-67 PI, tumor diameter, location, necrosis, and peritumoral edema [15–18]. Specifically, prognosis worsens with increasing pathological grade, as that indicates heightened tumor cell proliferation. Furthermore, Khanna et al. [19] used radiomics, a form of machine learning, to profile preoperative meningioma Ki-67 PI. The resultant predictions were successfully applied as a guide for determining the extent of surgical resection and improved prognosis. Similarly, here we demonstrated that tumor grade and Ki-67 PI were statistically significant in predicting meningioma recurrence, but not independent risk factors. However, our findings do not support the existing hypothesis regarding the relationship between tumor size and postoperative recurrence. This hypothesis states that a larger tumor diameter is correlated with greater probability of necrosis and edema, leading to an increase in compression severity on the surrounding brain tissue. As a result, surgical resection becomes more difficult, and the risk of postoperative recurrence rises. The discrepancy between the hypothesis and our results may be attributable to the relatively small sample size. In addition, meningiomas are blood-rich tumors, and heterogenous contrast enhancement, which may be a risk factor for predicting meningioma recurrence. The most likely explanation is that the heterogenous contrast enhancement may indicate the presence of populations of cells with different degrees of malignancy within the tumor, which may have different growth rates and aggressiveness, increasing the risk of recurrence.

In this study, we manually sketched ROIs based on maximum tumor level. Although this method loses some tumor information, it is far less cumbersome than whole-tumor histogram analysis and thus is more practical for clinical diagnosis in hospitals of all levels [20]. The results of comparing ADC histogram parameters revealed that they all differed significantly between recurrence and non-recurrence groups. This outcome is in line with histopathological data showing that high-grade meningiomas exhibit higher mitotic activity and increased nuclear/cytoplasmic ratios of tumor cells. These characteristics in turn lead to lower ADC values and poor prognosis. Moreover, variance is valuable for assessing tumor heterogeneity, as it mainly responds to the degree of data dispersion in histogram parameters [21]. Indeed, we observed that variance was slightly higher in the recurrence group than in the non-recurrence group.

Cytotoxic CD8 + T cells are the most abundant immune cells in the meningioma TME. High-grade meningiomas are immunologically “cold tumors,” with few CD3+, CD8+, and PD-1 + TILs compared with grade 1 tumors, but an elevated number of regulatory T cells [22]. The presence of CD3 + and CD8 + TILs in meningiomas have a positive effect on recurrence free survival, whereas a high percentage of PD1 + T cell infiltration is associated with shorter PFS [23]. Here, we confirmed that the number of CD8 + T cells was significantly lower in the recurrence group than in the non-recurrence group. Likewise, Zhang et al. [3] found that high levels of cytotoxic TILs were associated with improved PFS. Similarly, an association between high CD8 + TIL levels and improved RFS has been observed in patients with non-small cell lung cancer [24, 25] and hepatocellular carcinoma [26]. High CD8 + TIL density is also linked to better prognosis in melanoma [27] and colorectal cancer [28]. Taken together, our results and previous research illustrate the potential of CD8 + TILs as biomarkers for predicting meningioma recurrence.

Our findings suggest that ADCp1 and CD8 + T cells are independent risk factors for meningioma recurrence. This study is the first to integrate imaging, and TME data in a nonogram for predicting postoperative meningioma recurrence. Our model serves as an intuitive visual tool with demonstrable reliability and accuracy, while being an improvement to previous attempts. For example, a prior nomogram was built from clinical data to predict grade 2–3 meningiomas and measure meningioma prognosis [29, 30]. Similarly, Mo et al. [31] constructed a nomogram using independent predictors such as tumor size, Ki-67 index, and resection extent to stratify the recurrence risk of meningiomas. In contrast, we included all meningioma grades to generate a model with broader adaptations. Most importantly, our nomogram incorporated TME data on CD8 + T cells, better reflecting the heterogeneity of meningiomas and thus increasing predictive accuracy.

This study had several limitations. First, this was a single-center retrospective study with a small sample size. Our model must be validated with external data from prospective, large sample-size studies in the future. Secondly, because CD8 + T cells were included, we selected only tumor samples with the most recent paraffin sections, resulting in a shorter overall follow-up time for the patients enrolled. Recent studies have revealed that other TME factors (e.g., CD4 + T cells and PDL-1) are also strongly associated with meningioma recurrence. Thus, these variables should be included in future model-based research to further enhance nomogram predictive efficacy. Finally, for simplicity and feasibility, we chose ROIs based on maximum tumor level, potentially excluding portions of the tumor. Methods such as whole-tumor histogram analysis or machine learning should be explored in future studies to obtain a comprehensive view of the tumor.

## Conclusion

In summary, ADCp1 and CD8 + T cells may be potential in vivo biomarkers for predicting meningioma recurrence, providing clinicians with a basis for guidance in personalised treatment and postoperative follow-up of meningioma patients.

### Electronic supplementary material

Below is the link to the electronic supplementary material.

Supplementary Material 1

## Data Availability

The datasets used and/or analysed during the current study are available from the corresponding author on reasonable request.

## Abbreviations

MRI: Magnetic resonance imaging
ADC: Apparent diffusion coefficient
WHO: World Health Organization
CNS: Central Nervous System.
DWI: Diffusion-weighted imaging
TIL: Tumour infiltrating lymphocyte.
PFS: Progression-free survival
RFS: Recurrence-free survival
ROI: Region of interest
PI: Proliferation index
ICC: Interclass correlation coefficient
CI: Confidence intervals
HR: Hazard ratio
ROC: Receiver operating characteristic
AUC: Area under the curve
T1C: Contrast enhanced T1-weighted imaging
T2WI: T2-weighted imaging
T1WI: T1-weighted imaging
WSI: Whole Slide Image
TME: Tumour microenvironment
