# Supplementary material for: MRI features and tumor-infiltrating CD8 + T cells-based nomogram for predicting meningioma recurrence risk
Source: Cancer Imaging. 2024 Jun 28;24:79. doi: 10.1186/s40644-024-00731-6 (PMC11212175; doi:10.1186/s40644-024-00731-6)
Supplement: Supplementary file 1 — Supplementary Material 1 [file 40644_2024_731_MOESM1_ESM.docx]

Fig S1


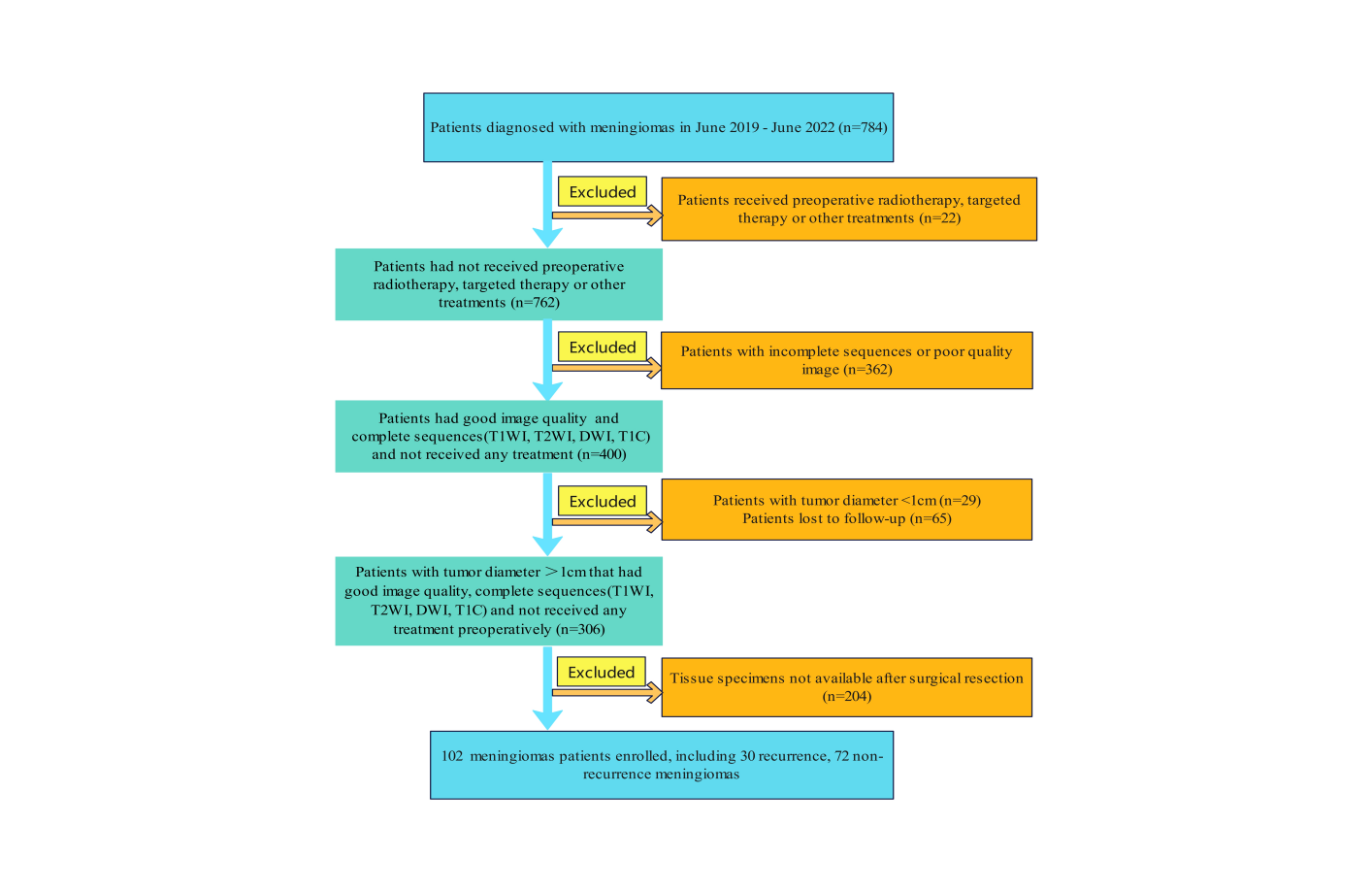


**Fig S1:** Flowchart of the patients selection process.

Fig S2


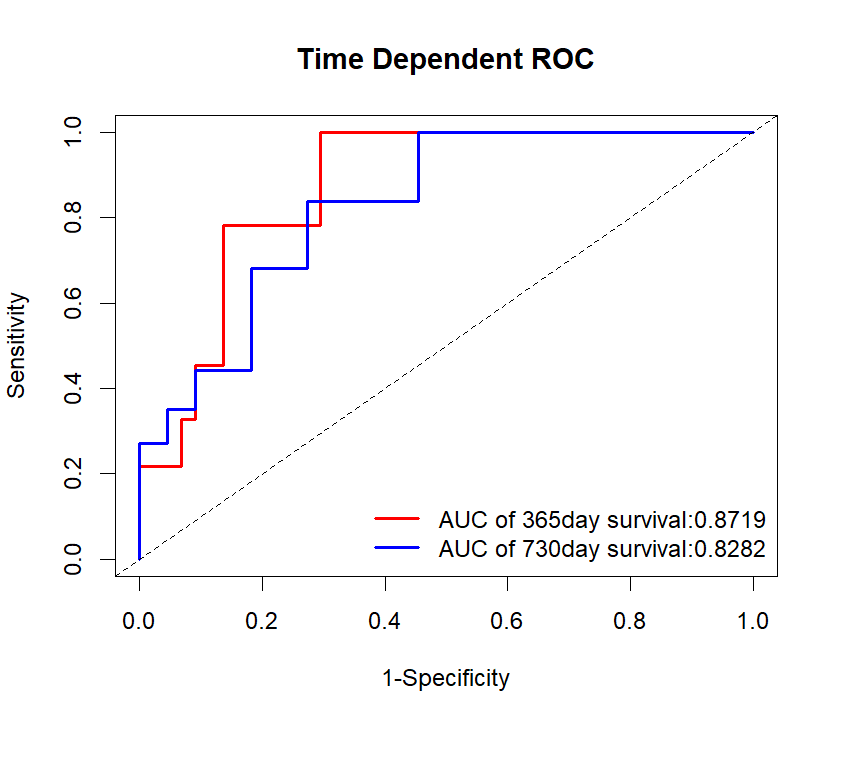

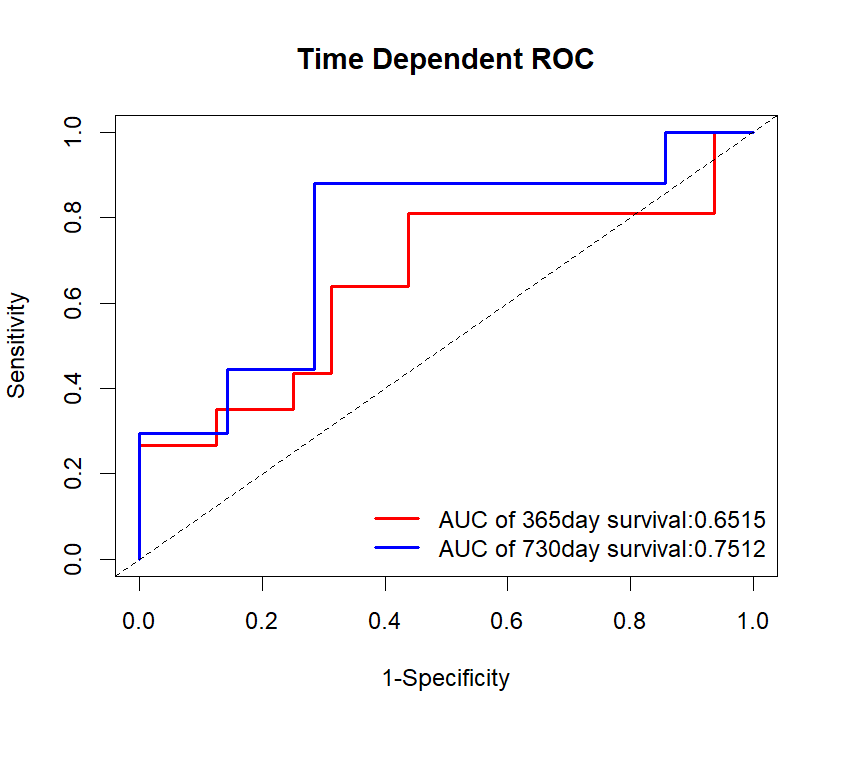


B

A

**Fig S2:** ROC curves for nomogram prediction of 1-year (A) and 2-year (B) progression-free recurrence of WHO grade 1 and WHO grade 2 meningiomas.
